# Numerical analysis of low-frequency electromagnetic field effects from three-phase transformer on coronary stents and cardiac tissues

**DOI:** 10.1371/journal.pone.0340031

**Published:** 2026-01-30

**Authors:** Rui Tian, Jia-Yi Luo, Mai Lu, Jing-Jing Cui

**Affiliations:** 1 Key Laboratory of Opto-Electronic Technology and Intelligent Control, Ministry of Education, Lanzhou Jiaotong University, Lanzhou, China; 2 China Railway First Survey and Design Institute Group Co., Ltd., Xi’an, Shaanxi, China; Gwangju Institute of Science and Technology, KOREA, REPUBLIC OF

## Abstract

The widespread clinical adoption of novel magnesium alloy coronary stents, combined with increasing densification of urban power transmission infrastructure, highlighted a significant research gap regarding the effects of power-frequency electromagnetic fields (EMFs) on these implants. This study employed field-circuit coupling numerical methods to simulate electromagnetic field exposure in simulated patients with implanted coronary stent positioned at various locations near a 200kVA three-phase transformer. The analysis focused on the distribution patterns of induced electromagnetic fields within both cardiac tissues and the stent, as well as the resultant Ampere forces acting on the stent. The results showed that the simulated patient directly beneath the three-phase transformer was exposed to the maximum electromagnetic radiation, but the magnetic flux density (***B***_***max***_) and the induced electric field intensity (***E***_***max***_) of the cardiac tissue were lower than the public exposure limits of the International Commission on Non-Ionizing Radiation Protection (ICNIRP). The ***B***_***max***_ and ***E***_***max***_ of the stent at the same position were 1.245 *μ*T and 5.086 × 10^−4^ mV/m, respectively. The maximum Ampere force density of the stent in the Y- axis (perpendicular to the coronal plane) was 3.714 × 10^−6^ N/m^3^. The above findings indicate that, under the conditions of this simulation, a 200 kVA power transformer exerts minimal interference on the magnesium alloy stent and cardiac tissues. The magnetic flux density and induced electric field in the heart tissues, as well as the Ampere force acting on the magnesium alloy stent, all remain within established safety limits.

## 1. Introduction

Transformers are core components of modern power systems. With the acceleration of urbanization in China, the increasing density of transmission and transformation facilities has significantly reduced the distance between the public and sources of electromagnetic radiation, leading to a notable rise in exposure to power-frequency electromagnetic environments. Existing studies indicate that extremely low-frequency electromagnetic fields may, under certain conditions, affect human heart rate and cognitive function, such as causing bradycardia and delayed cognitive processing [1]; however, other research suggests that long-term exposure to extremely low-frequency magnetic fields can enhance the viability of human lymphoblastoid cells without evidence of DNA or chromosomal damage [2]. Additionally, a uniform 60 Hz electromagnetic field can promote the proliferation of both normal and cancerous human cells, which may facilitate tissue repair and wound healing but could also accelerate tumor formation [3]. It can be seen that exposure to extremely low-frequency electromagnetic fields has a dual nature: under specific conditions, it may pose potential health risks while also offering therapeutic benefits. This requires particular attention from vulnerable populations, especially the growing number of simulated patients with cardiovascular diseases. As the leading cause of death globally, cardiovascular disease is commonly treated with stent implantation, with millions of stents deployed worldwide each year [4]. Among these, novel magnesium alloy stents have emerged as a promising alternative for coronary heart disease treatment, as they avoid long-term complications associated with permanent implants [5–6]. However, as metallic implants, cardiac stents inevitably become subjects of electromagnetic induction when exposed to power-frequency magnetic fields generated by transmission and distribution facilities. Previous researchers have found that carotid artery stents can cause a significant increase in local SAR (Specific Absorption Rate) under radiofrequency (RF) electromagnetic field exposure [7], and electromagnetic leakage from wireless energy transfer systems in electric vehicles may also pose potential electromagnetic safety risks to simulated patients with coronary stents [8]. Meanwhile, clinical observations indicate that stents may deform or fracture after implantation, though the causes are often multifactorial [9–10]. Among various potential factors, the impact of power-frequency electromagnetic fields on stent safety remains insufficiently studied. Given the ubiquitous nature of electromagnetic fields in daily life, the safety of cardiac stents under electromagnetic exposure has become an urgent public health issue.

Relevant studies indicate that although experiments on cadaver models under routine environmental electromagnetic exposure did not result in significant temperature rise of metallic implants, they did not rule out other potential biological effects [11]. Numerical simulations by Shah et al. revealed that a 6.78 MHz/50W wireless charging system markedly alters SAR distribution around micro-implants [12], Mengxi Zhou et al. experimentally demonstrated that high-intensity low-frequency electric fields may induce functional impairments in implanted devices [13]. Conversely, Barz et al. concluded that therapeutic magnetic fields and power line magnetic exposure levels appeared safe for simulated patients with coronary stents [14]. M. Olteanu et al. numerically simulated the effects of an 835 MHz radiofrequency field on the SAR and temperature around stents, finding that the maximum temperature rise in peri-stent tissue was below 1°C, while the local SAR values exceeded standard limits [15]. To mitigate RF-induced heating during magnetic resonance imaging (MRI), Dawei Li et al. proposed a novel segmented esophageal stent design [16]. Collectively, high-frequency studies have emphasized SAR and thermal assessments, whereas low-frequency research primarily has examined magnetic field effects while largely neglecting induced electric fields in metallic conductors and Ampere forces from eddy current-magnetic field interactions—factors that may contribute to implant deformation.

Due to ethical and technical constraints, direct measurement of electromagnetic exposure levels in specific areas of the human body remains a significant challenge. Existing studies often opt for experiments conducted in animal models [17] or in vitro cell cultures [18–19]. Some research has also attempted to analyze far-field radiation characteristics by implanting antennas in animals [20], or to monitor changes in tissue dielectric properties caused by physiological variations using implantable biosensors [21], however, these methods are invasive and difficult to apply directly in humans. Therefore, numerical simulation has become an important method for assessing electromagnetic dose values within the human body. In this study, we first established a three-dimensional electromagnetic field model of a 200 kVA/50 Hz three-phase power transformer using the finite element method, while constructing a magnesium alloy stent model based on a digital human. Subsequently, we applied circuit excitation to the transformer’s primary and secondary coils, using field-circuit coupling numerical methods to calculate the spatial electromagnetic field distribution. Finally, we analyzed the distribution characteristics of induced electric and magnetic fields in both cardiac tissue and the magnesium alloy stent at different exposure positions beneath the transformer. Furthermore, we calculated the Ampere forces acting on the magnesium alloy coronary stent under electromagnetic coupling effects, providing valuable insights for analyzing potential causes of deformation in coronary stents.

## 2. Physical model

### 2.1 Magnesium alloy coronary stent model

**Fig 1:**
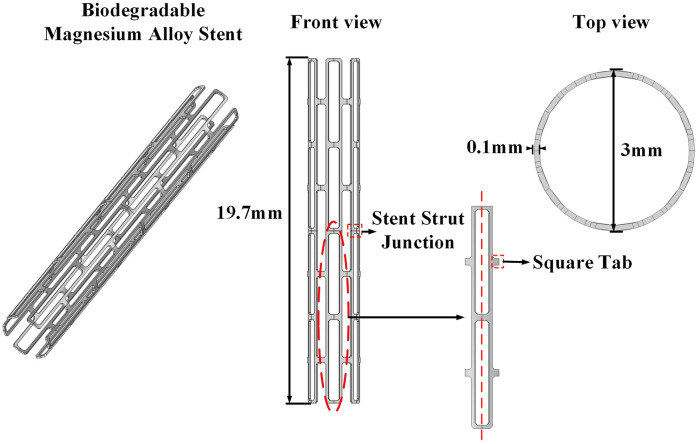
Magnesium alloy coronary stent model.

To ensure coronary lumen patency, reduce restenosis rates, and enhance the mechanical stability of coronary stent, the magnesium alloy stent adopted in this study features the configuration illustrated in Fig 1. The stent comprises 24 interlocking curved units forming a symmetrical cylindrical structure. One group of units contains square protrusions at the left 1/4 and 3/4 positions, as well as at both ends and the midpoint on the right side, while the other group exhibits a mirror-symmetric design. These two sets of components are interconnected in an alternating pattern, forming a uniform-diameter hollow cylinder through coordinated arrangement. The key dimensional parameters include a total length of 19.7 mm, an external diameter of 3 mm, and a wall thickness of 0.1 mm.

The stent is fabricated from AZ31 magnesium alloy, with Young’s modulus of 43.5GPa, Poisson’s ratio of 0.35, and density of 1.77 g/cm^3^. This material is modeled as a homogeneous, isotropic elastoplastic solid [22]. The coronary stent geometry is shown in Fig 1.

### 2.2 Human body and heart model

**Fig 2:**
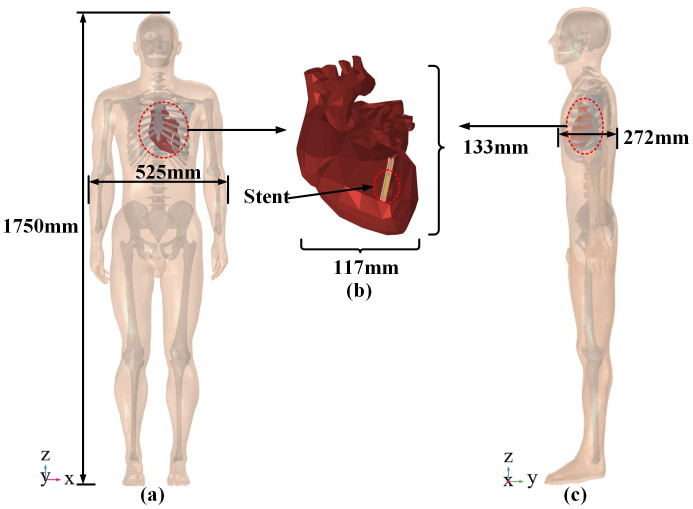
Human body and heart relative position model. (a) Front view of the human body. (b) Heart model. (c) Side view of the human body.

The human model is a 175 cm tall adult male, with the heart located left-inferior to the thoracic midline. The heart measures 133 mm in longitudinal diameter and 117 mm in transverse diameter [23], as illustrated in Fig 2. Since this study focuses on the electromagnetic exposure of the heart and stents, the relevant parameters of the body were simplified by taking the unweighted arithmetic mean of selected tissues (muscle, skin, cortical bone). The relative permittivity and electrical conductivity parameters at 50 Hz were obtained from the publicly accessible online database provided by the Institute of Applied Physics of the Italian National Research Council. This database, which implements the fourth-order Cole-Cole model proposed by Gabriel et al. [24–26], enables the querying of tissue properties across a specified frequency range. The dielectric properties of human tissues are listed in Table 1.

**Table 1:** Parameters of human tissues [27].

Human tissue	Relative dielectric constant	Conductivity(S/m)	Relative permeability
Muscle	17719000	0.23329	1
Skin	1136	0.0002	1
Cortical bone	8867.8	0.020055	1
Myocardium	1472800	0.019555	1
Fat	8664600	0.082729	1
Vascular wall	8095800	0.26115	1
Blood	5259.9	0.7	1

**Fig 3:**
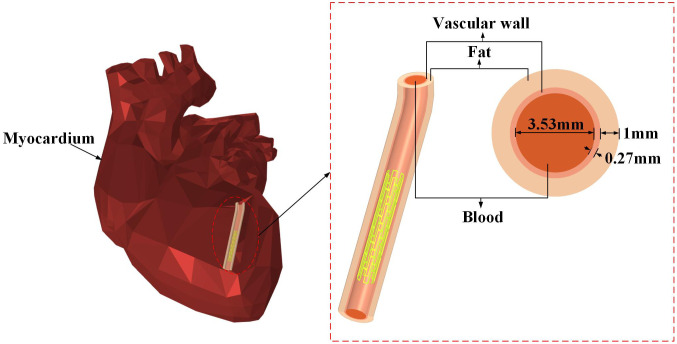
Cardiac model with implanted magnesium alloy stent.

The left coronary artery, originating from the aortic left coronary sinus and running downward along the coronary sulcus, functions as the heart’s main blood supply channel for the left ventricle and surrounding myocardium. With an internal diameter of 3.53 mm and wall thickness of 0.27 mm [28], the blood vessel is surrounded by a 1 mm-thick layer of epicardial fat to better simulate physiological conditions [29]. The heart model, with no further subdivisions, shows the stented coronary artery and vascular structure in Fig 3.

### 2.3 Three-phase transformer model

**Fig 4:**
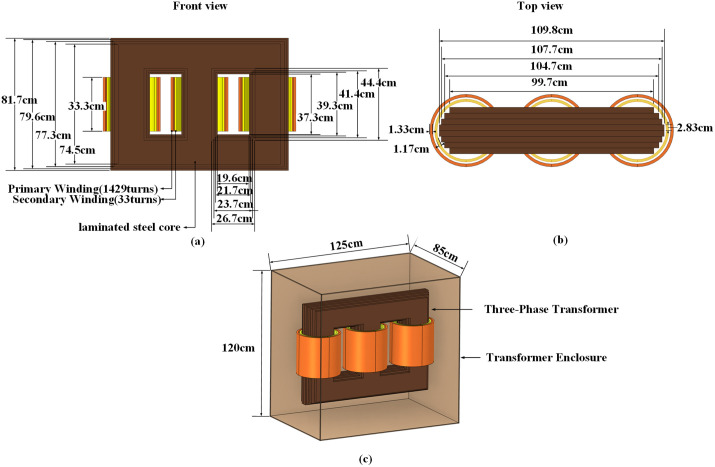
Transformer model. (a) Front view. (b) Top view. (c) Overall Diagram.

**Fig 5:**
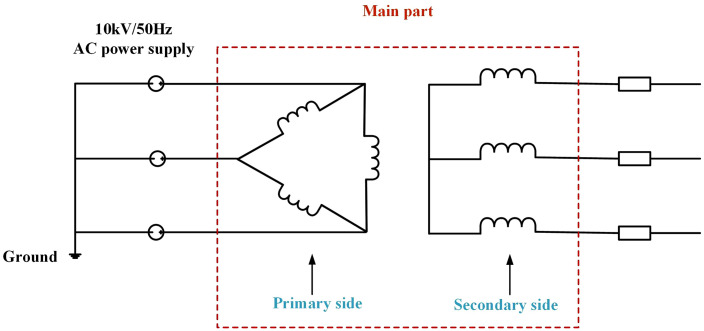
Circuit schematic diagram of the three-phase power transformer.

The three-phase three-limb power transformer model is designed according to mainstream urban distribution network equipment parameters, with a rated apparent power of 200 kVA and operating frequency of 50 Hz. It achieves power-frequency voltage transformation from 10 kV to 400 V. The transformer structure comprises three primary components: core, windings, and metal enclosure. The laminated silicon steel core features primary and secondary windings arranged radially along each limb in stratified layers, where primary coils occupy outer layers and secondary coils inner layers. The 2 mm-thick enclosure utilizes stainless steel [30]. Fig 4 details the transformer structure, while Fig 5 shows its electrical schematic: primary windings adopt a delta connection, and secondary windings a star connection.

### 2.4 Integrated model

**Fig 6:**
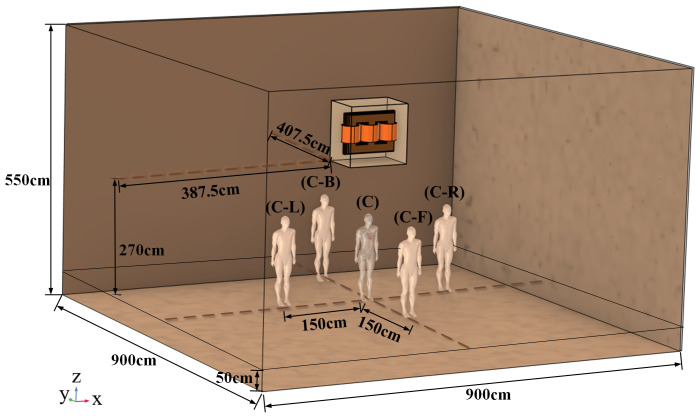
Entire model.

This study employs the Magnetic Fields (MF) module of the commercial multiphysics simulation software COMSOL Multiphysics 6.2 to investigate the electromagnetic exposure of the human body and cardiac stents under power-frequency electromagnetic fields. In order to accurately assess the electromagnetic impact from the three-phase transformer and to simulate real-life scenarios, electromagnetic exposure simulations are performed at a height of 270 cm above the ground for the three-phase transformer [31]. A rectangular air domain with dimensions of 900 cm × 900 cm × 550 cm is used as the computational space, which completely wraps the human body model and the transformer system. The integrated human body-transformer model is illustrated in Fig 6.

**Fig 7:**
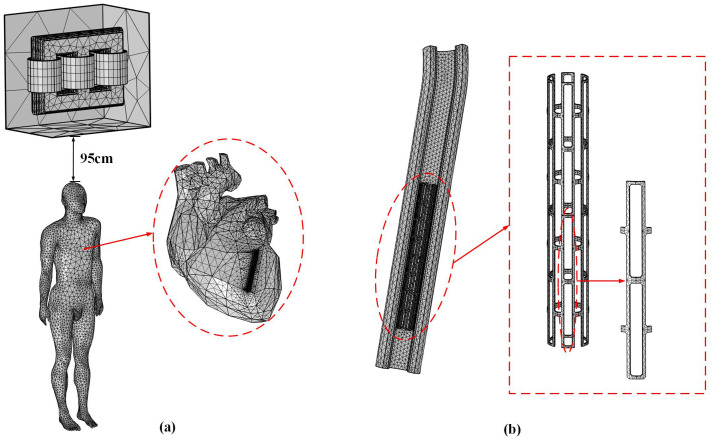
Model grid division diagram. (a) Overall model. (b) Blood vessel and stent model.

Combining the initial and boundary conditions, we performed a refined meshing of the computational domain. After mesh optimization, the model attained 2,509,937 degrees of freedom and was solved using the built-in direct solver (PARDISO) in COMSOL, requiring approximately 2.5 hours on a computer with 16 GB of RAM. The meshing model is shown in Fig 7.

## 3. Governing equations

### 3.1 Low-frequency magnetic field control equation

Current density in three-phase transformer windings:


$$\begin{array}{c} J_{e}=\frac{nI_{coil}}{S}e_{coil} \end{array}$$ (1)

where $J_{e}$ is the externally generated current density of the coil (A/m^2^), n is the number of turns in the coil, $e_{coil}$ denotes the unit vector in current direction, $I_{coil}$ indicates the conductor current (A), S is the cross-sectional area of the coil (m^2^).

Under power frequency conditions (50 Hz quasi-static field), the magnetic vector potential A (Wb/m) can be obtained from the winding current density:


$$\begin{array}{c} \Omega :\nabla \times v\nabla \times A=J_{e} \end{array}$$ (2)

$$\begin{array}{c} \Gamma_{\text{1}}:A=A_{0} \end{array}$$ (3)

Ω represents the solution domain, $v=\;\frac{\text{1}}{\mu }$ is the reluctivity with μ being the permeability. Equations (3) define the Type I boundary condition, where $A_{0}$ is the initial value of the magnetic vector potential. Magnetic flux density ***B*** (T) and electric field intensity ***E*** (V/m) generated by the three-phase transformer are derived from electromagnetic field definitions:


$$\begin{array}{c} B=\nabla \times A \end{array}$$ (4)

$$\begin{array}{c} \nabla \;\cdot \;A=\;\text{0} \end{array}$$ (5)

$$\begin{array}{c} E=\;-j\omega A \end{array}$$ (6)

In the equation, ω represents the angular frequency (rad/s). In practical applications, the magnetic flux density ***B*** (T) is influenced by the surrounding medium. The biological tissues considered in this study are all treated as isotropic, with their constitutive relation given by Equation (7):


$$\begin{array}{c} B=\mu H \end{array}$$ (7)

where **H** is the magnetic field intensity (A/m). Additionally, the induced electric field satisfies Equation (8), the magnetic field satisfies Equation (9):


$$\begin{array}{c} \nabla \times E=-j\omega B \end{array}$$ (8)

$$\begin{array}{c} \nabla \cdot B=\;\text{0} \end{array}$$ (9)

### 3.2 Force analysis

According to electromagnetic induction principles, when a magnesium alloy stent is exposed to an alternating magnetic field, an induced current is generated within the stent. The induced current density is given by Equation (10):


$$\begin{array}{c} J_{in}=\sigma E=-\sigma j\omega A \end{array}$$ (10)

where $J_{in}$ in represents the induced current density and σ is the electrical conductivity of the stent.

The current in the magnesium alloy stent experiences Ampere forces when subjected to the magnetic field, with its magnitude and direction determined by Equation (11):


$$\begin{array}{c} F_{v}\;=\;J_{in}\;\times \;B \end{array}$$ (11)

### 3.3 Boundary conditions

**Fig 8:**
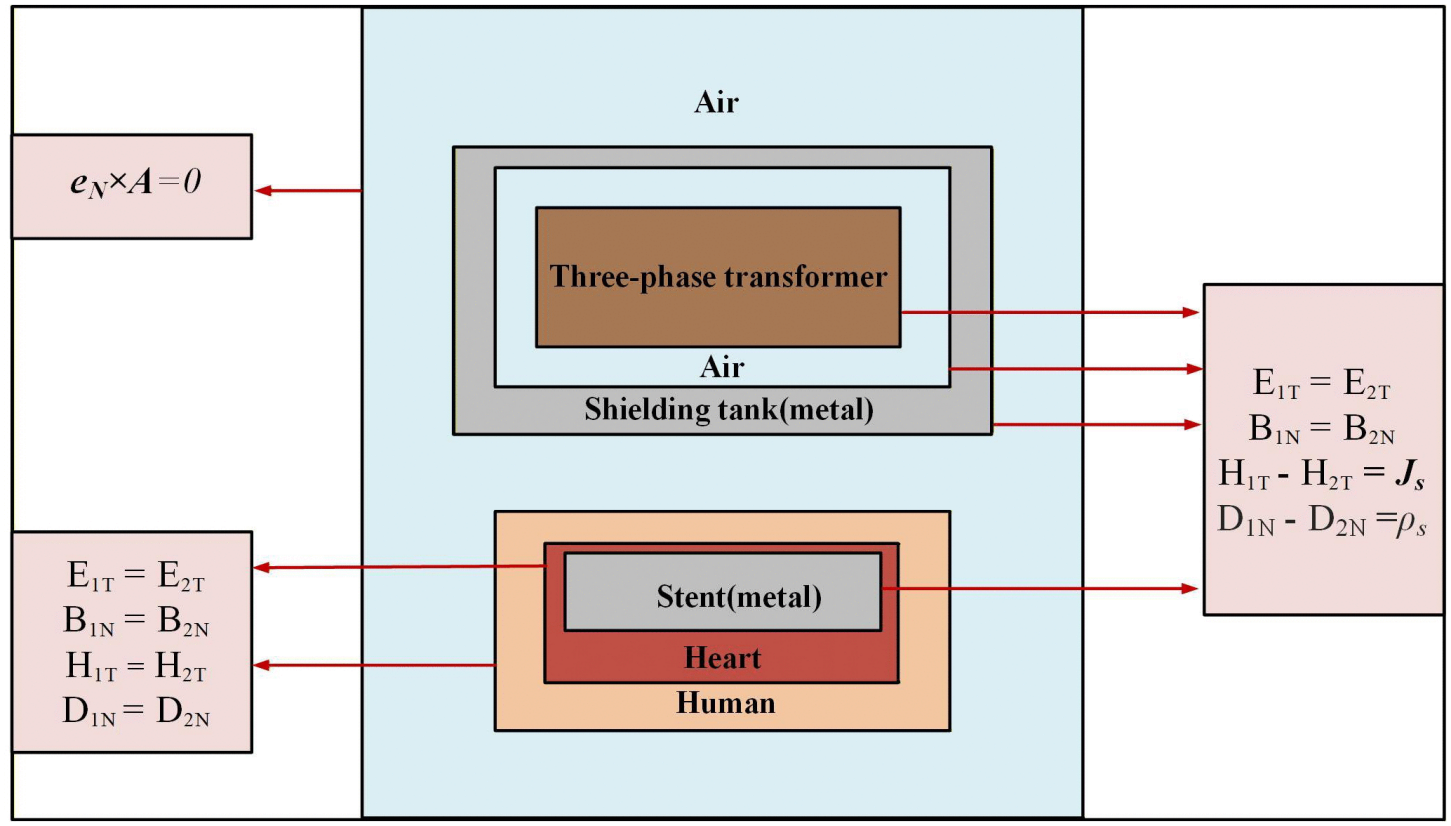
Boundary conditions.

The boundary conditions are illustrated in Fig 8. In the figure, the subscript N denotes the normal component perpendicular to the interface, the subscript T indicates the tangential component parallel to the interface. **J**_**s**_ and $\rho_{s}\;$represent the surface current density and free charge density on the metal surface, respectively, and are applicable to transformer, shield and stent.

### 3.4 Method validation

To better demonstrate the accuracy of the low-frequency magnetic field exposure assessment method proposed in this study, we replicated the conditions described in References [32] to analytically calculate the interaction force between two current-carrying straight wires (each 30 cm long and spaced 1 cm apart) carrying 6 A of current.

According to Ampere’s circuital law, the magnetic flux density at a distance d from a current-carrying straight wire is given by:


$$\begin{array}{c} B=\frac{\mu_{\text{0}}I}{\text{2}\pi d} \end{array}$$

where the vacuum permeability μ_0_ ≈ 1.26 × 10^-6^N/A^2^, I is the current intensity (A), and d is the distance between the axes of the two wires (m). Based on the Ampere’s force law, the force acting on a wire in a magnetic field is:


$$\begin{array}{c} F=BLI=\frac{\mu_{\text{0}}L}{\text{2}\pi d}I^{\text{2}} \end{array}$$

L represents the length of the current-carrying wire (m). The calculated interaction force between the current-carrying wires is

***F***_**12**_ = ***F***_**21**_ = 2.160 × 10^-4^ N

**Fig 9:**
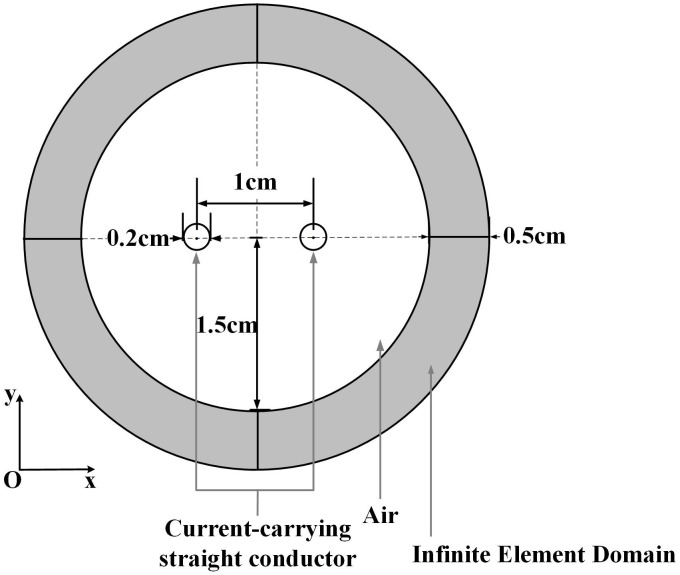
Two-dimensional model of current-carrying wires.

The numerical method is applied to calculate the Ampere force acting on the current-carrying straight wires mentioned above. The model is shown in Fig 9, with an air gap radius of 1.5 cm and an infinite element domain of 0.5 cm thickness surrounding it.

The simulated Ampere forces were:


$$F_{12}\;=\;\sqrt{F_{1x}^{2}+F_{1y}^{2}}=\text{2}.\text{1}\text{6}\text{0}\times {\text{1}\text{0}}^{-\text{4}}N$$

$$F_{21}\;=\;\sqrt{F_{2x}^{2}+F_{2y}^{2}}=\text{2}.\text{1}\text{6}\text{0}\times {\text{1}\text{0}}^{-\text{4}}N$$

Magnetic-mechanical coupled numerical simulations reveal minimal discrepancies between the calculated interaction forces of current-carrying wires and their theoretical analytical solutions.

**Fig 10:**
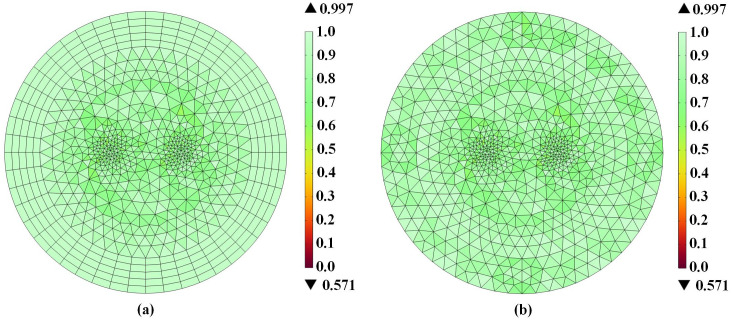
Meshing of the two-dimensional model. (a) Mapped meshing for infinite domains. (b) Free triangular meshing.

As mesh quality significantly impacts numerical simulation accuracy, we systematically evaluated grid discretization schemes and density effects. Fig 10 compares two meshing strategies (a) and (b), and Table 2 presents corresponding force calculations.

**Table 2:** Mesh density validation.

Meshing scheme	Number of Mesh Elements	F_12_	Error	F_21_	Error
a	1078	2.158 × 10^-4^N	0.09%	2.158 × 10^-4^N	0.09%
b	1392	2.156 × 10^-4^N	0.18%	2.156 × 10^-4^N	0.18%
a	2790	2.159 × 10^-4^N	0.05%	2.159 × 10^-4^N	0.05%
a	5524	2.159 × 10^-4^N	0.05%	2.159 × 10^-4^N	0.05%
a	9176	2.160 × 10^-4^N	0.00%	2.160 × 10^-4^N	0.00%
a	14104	2.160 × 10^-4^N	0.00%	2.160 × 10^-4^N	0.00%

Numerical simulations and analytical results from the literature consistently demonstrate that enhanced mesh refinement yields improved computational accuracy. Across various meshing configurations, the maximum errors in the mutual forces were 0.09% for configuration (a) and 0.18% for configuration (b). These results confirm that the low-frequency electromagnetic simulation method employed in this study can accurately calculate the electromagnetic forces exerted by the three-phase transformer on the coronary stent.

## 4. Results and discussion

**Fig 11:**
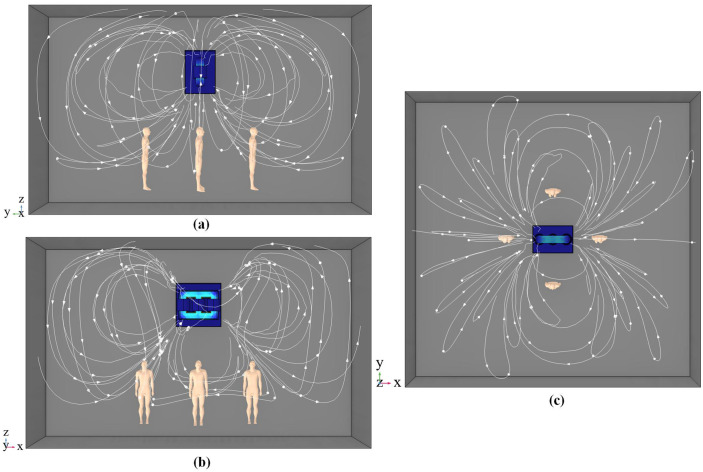
Magnetic flux line distributions. (a) Front view. (b) Side view. (c) Top view.

Due to article length constraints and our primarily focus on the electromagnetic exposure effects on coronary stent, this paper only presents electromagnetic field data for cardiac tissues (myocardium, fat, blood vessels, blood) and magnesium alloy stent when the human body is positioned at different locations beneath the three-phase transformer. Fig 11 shows the macroscopic magnetic flux density generated by the transformer in space, with white lines indicating the spatial magnetic field distribution. Fig 11 also reveals denser magnetic field lines along the x-axis compared to the y-axis below the transformer, indicating stronger magnetic field intensity in the x-direction. Meanwhile, directly beneath the transformer, the human body is closest to the source and is traversed by the greatest number of magnetic flux lines. In this location, the simulated patient exhibits the highest magnetic flux density.

### 4.1 Magnetic field distribution

#### 4.1.1 Magnetic field distribution in cardiac tissues.

**Fig 12:**
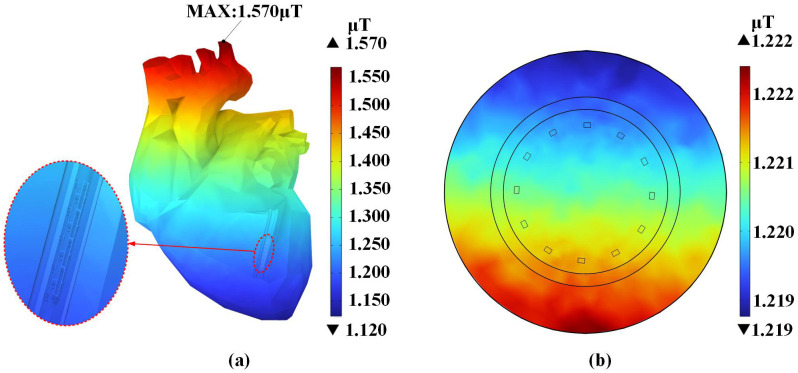
Magnetic flux density distribution at position (C) (a) Frontal view of the heart. (b) Cross-section of blood vessel.

Fig 12 illustrates the distribution of the magnetic flux density within the cardiac tissues when the body is located at position (C).

Fig 12 demonstrates that the peak magnetic flux density in the heart occurs at the top region of the aorta. Due to the non-ferromagnetic properties of the magnesium alloy, the implant does not induce localized magnetic field distortion.

**Fig 13:**
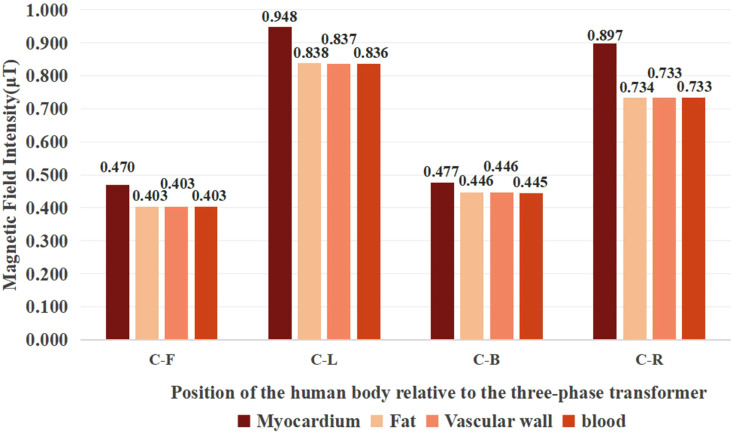
Histograms of the distribution of magnetic field strength in various tissues of the cardiac tissues in different positions.

Fig 13 presents the distribution of the magnetic flux density of the myocardium, fat, vascular wall and blood when the human body is in different positions (C-F), (C-L), (C-B) and (C-R).

Fig 13 indicates that, the magnetic flux density of all cardiac tissues reaches peak values at positions (C-L), with the exception of position (C). Notably, the myocardium consistently shows the highest magnetic flux density at every position beneath the three-phase transformer. Combined with Fig 11, it can be observed that the magnetic flux density of various cardiac tissues along the x-axis (positions (C-L) and (C-R)) is generally higher than that along the y-axis (positions (C-F) and (C-B)).

#### 4.1.2 Magnetic field distribution in magnesium alloy stent.

**Fig 14:**
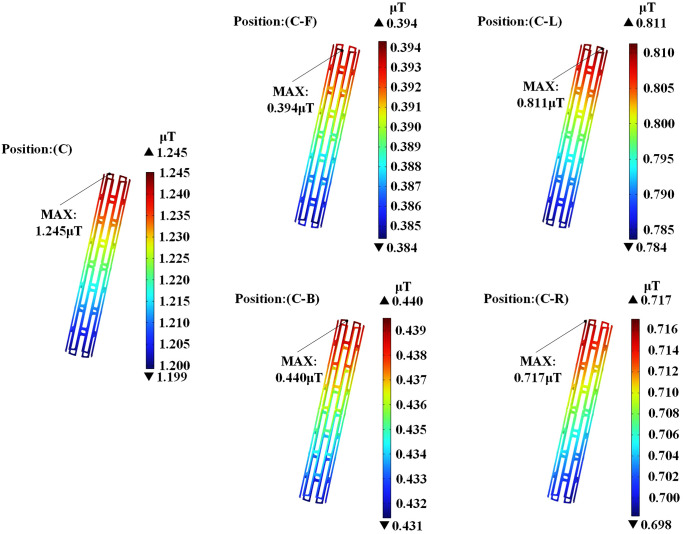
Magnetic flux density distribution in the magnesium alloy stent.

Fig 14 presents the magnetic flux density distribution of the magnesium alloy stent across different positions. The stent attains its peak magnetic flux density at position (C). At any position below the transformer, the stent’s peak magnetic flux density always occurs at its uppermost part.

### 4.2 Induced electric field distribution

#### 4.2.1 Induced electric field distribution in cardiac tissues.

**Fig 15:**
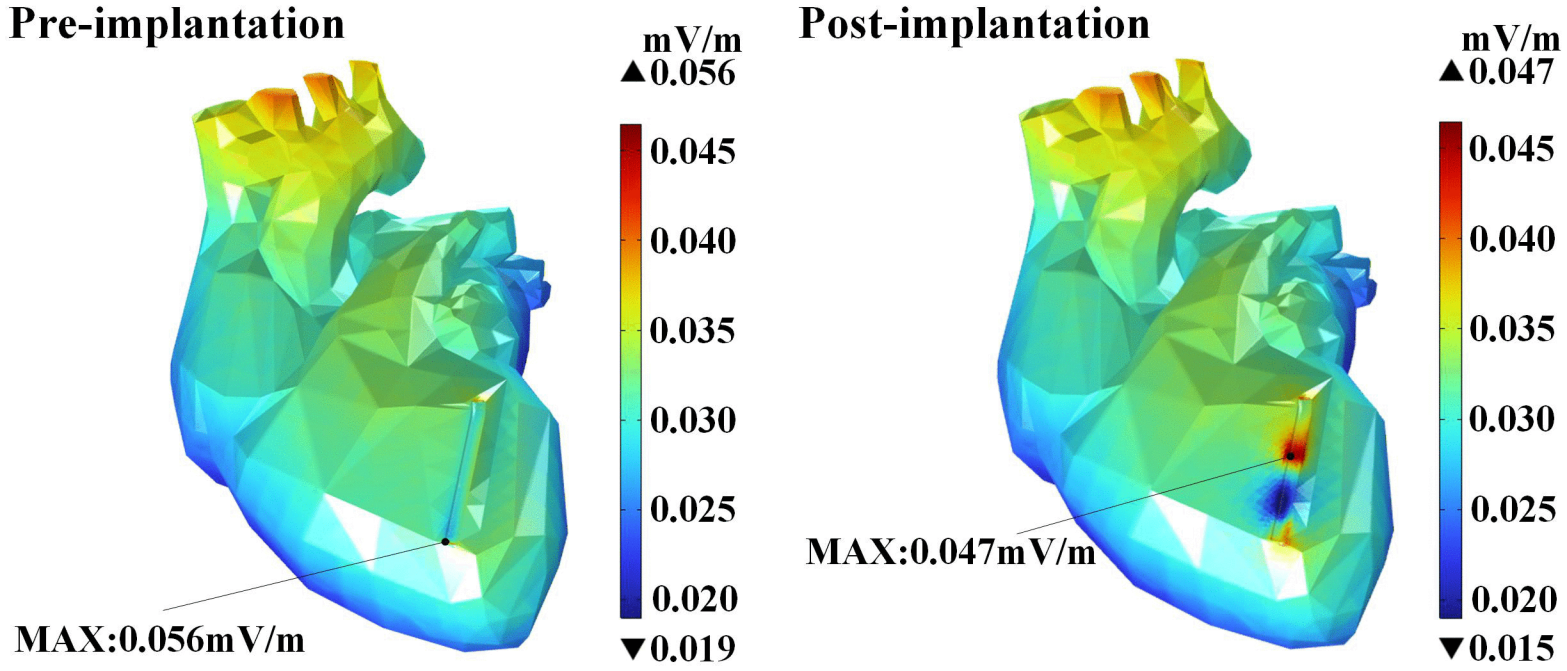
Induced electric field distribution in myocardium at position (C) (a) Pre-implantation. (b) Post-implantation.

The cardiac tissues (myocardium, fat, vascular wall, and blood) and the magnesium alloy stent show distinct electrical properties. By the Faraday’s law, an induced electric field forms inside the stent, leading to eddy currents. Via secondary electromagnetic responses, these eddy currents further alter the local induced electric field distribution in the cardiac tissues. Fig 15 shows the induced electric field distribution in the myocardium before and after stent implantation.

**Fig 16:**
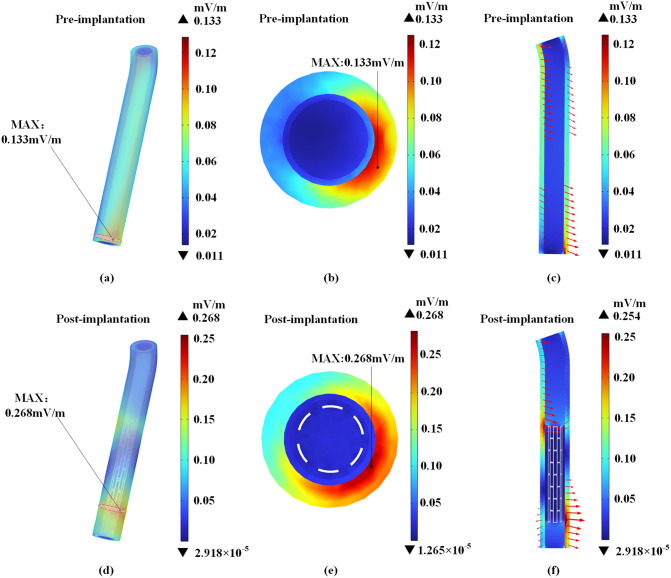
Distribution of induced electric field in blood vessel located at position (C). Without stent implantation: (a) Overall view, (b) Cross-sectional view, (c) Longitudinal section. With stent implantation: (d) Overall view, (e) Cross-sectional view, (f) Longitudinal section.

The local induced electric field intensity of the blood vessel will change significantly after the coronary stent is implanted. Fig 16 presents the induced electric field distribution of blood vessel (fat, vascular wall, and blood) at position (C) before and after the magnesium alloy stent is in place.

As shown in Fig 16, the maximum induced electric field value of the blood vessel occurs in the fat layer, which is about 2.02 times higher than the maximum value before the stent is implanted. In regions where the normal component of current dominates at tissue boundaries, the current density remains continuous. According to Ohm’s law, this results in a stronger induced electric field in fat due to its lower conductivity. In areas where the tangential component of current prevails, the electric field strength remains approximately equal on both sides of the interface. In Fig 16(c) and 16(f), the red arrows indicate the direction of current flow, with their lengths proportional to the magnitude of the current density.

**Fig 17:**
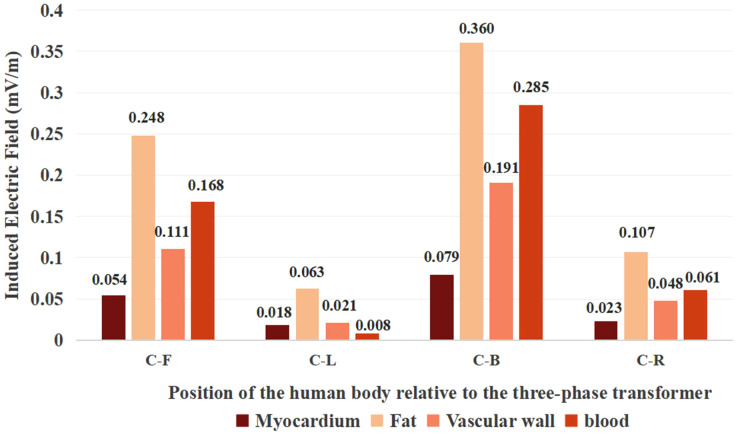
Histograms of the distribution of induced electric field strength in various tissues of the heart in different positions.

From the comparison in Fig 17, it can be seen that fat reaches the highest induced electric field strength in cardiac tissue at all positions, which is 4.56, 1.88, and 1.26 times higher than the maximum values in the myocardium, vascular wall, and blood, respectively.

#### 4.2.2 Induced electric field distribution in magnesium alloy stent.

**Fig 18:**
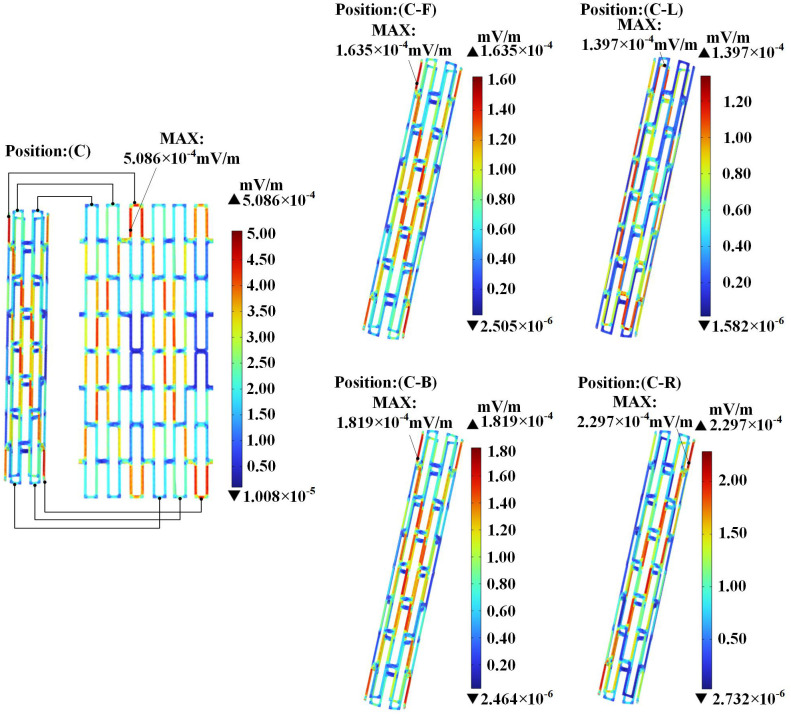
Induced electric field strength in the magnesium alloy stent.

Fig 18 shows the induced electric field distribution within the coronary stent in simulated patients that were positioned at different locations below the transformer. Due to the geometric structure of the stent, the induced electric field exhibits a non-uniform distribution pattern within the stent. Notably, the overall intensity of the induced electric field in the stent at position (C) is higher compared to other locations. When the stent at position (C) is unfolded into a planar view for observation, the induced electric field demonstrates a tendency toward symmetrical distribution.

### 4.3 Ampere forces of magnesium alloy stent

**Fig 19:**
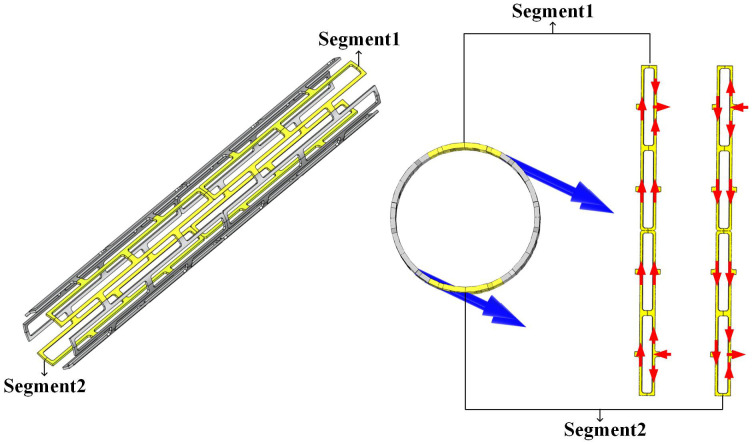
Magnetic field and current distributions in the opposing stent segments.

This section discusses the mechanical effects of low-frequency magnetic fields on the stent. Taking the patient positioned directly beneath the transformer as an example. Based on electromagnetic principles, the alternating magnetic field induces eddy currents in the magnesium alloy stent. Under the time-varying magnetic field, these eddy currents generate Ampere forces, the direction of which is determined by the left-hand rule. To clarify the force distribution in the stent structure, two sets of opposing stent segments (highlighted in Fig 19) were selected to analyze their magnetic field and current distributions.

As shown in Fig 19, in these two sets of opposing stent segments, while the magnetic field direction remains identical, the eddy current directions are opposing. Consequently, the Ampere forces acting on the opposing stent segments exhibit opposite directions, leading to mutual cancellation of most forces within the overall stent. In Fig 19, the blue and red arrows denote the magnetic field direction and current direction, respectively.

**Fig 20:**
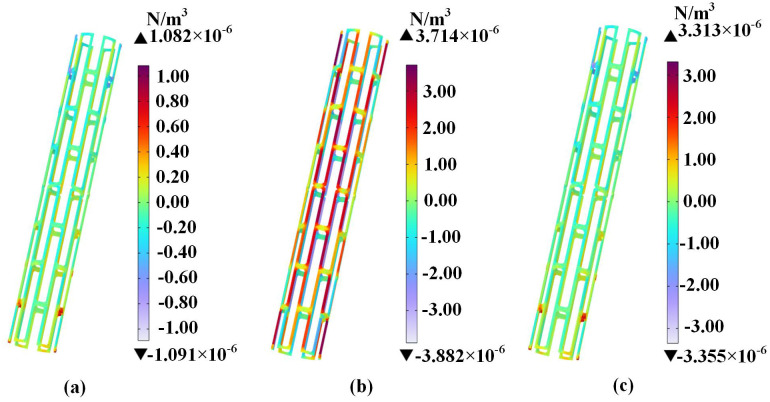
Ampere force distribution in the magnesium alloy stent. (a) x-axis; (b) y-axis; (c) z-axis.

The distribution of Ampere force density in all directions of the stent is shown in Fig 20.

Although the magnitude of the Ampere force density is relatively small, the non-uniform force distribution on the stent may lead to localized stress concentration. For instance, at the nodal junctions between stent, the localized stress can reach 2.579 × 10^−6^ Pa. Nevertheless, this value still significantly below the yield strength of magnesium alloys [33], indicating that electromagnetic forces cannot cause structural deformation in the coronary stent.

## 5. Conclusions

This study numerically analyzed the electromagnetic field exposure from 200 kVA/50 Hz three-phase power transformers in urban environments, focusing on the magnetic flux density and induced electric field intensity in both cardiac tissues and magnesium alloy stent of simulated patient, along with the resultant Ampere forces on the stent. In the assessment of human electromagnetic exposure safety, this study adheres to the public exposure limit standards specified in the *Guidelines for Limiting Exposure to Time-Varying Electric and Magnetic Fields (1 Hz to 100 kHz)* published by the International Commission on Non-Ionizing Radiation Protection (ICNIRP) [34]. Analysis of the simulation results leads to the following conclusions:

1. The cardiac tissue and magnesium alloy stent at position (C) showed peak magnetic flux density values of 1.570 *μ*T and 1.245 *μ*T, respectively. The maximum magnetic flux density consistently concentrated at the geometric apex in all tissues, exhibiting gradual attenuation along the longitudinal direction. The magnetic flux density at x-axis positions (C-L) and (C-R) beneath the three-phase transformer was significantly higher than at y-axis positions (C-F) and (C-B). In all cardiac tissues across every location, the magnetic flux density values remained well below the ICNIRP public exposure limit of 200 *μ*T.
2. From the distribution of the induced electric field, it is evident that the induced electric field within the cardiac tissues exhibited non-uniform distribution due to the distinct structures of biological tissues as well as differences in their dielectric constants and electrical conductivities. In cardiac tissue, the maximum induced electric field was observed in fat at position (C-B), while the stent’s maximum value was 5.086 × 10^−4^ mV/m at position (C). All values comply with the ICNIRP 400 mV/m public exposure guideline.
3. In the power-frequency alternating magnetic field, the stent was subjected to Ampere forces. The maximum value of the Ampere force density in the y-axis is 3.714 × 10^−6^ N/m³. The maximum local stress is 2.579 × 10^−6^ Pa, which is significantly lower than the yield strength of the magnesium alloy. This study suggests that under the simulated conditions, the electromagnetic field generated by a 200 kVA transformer exerts Ampere forces on the coronary stent that are substantially lower than the yield strength of the magnesium alloy, indicating a negligible risk of structural deformation. These results provide important clinical reference data, contributing to the assessment of electromagnetic safety for patients with coronary stents in typical urban environments.

In real-world scenarios, the behavior and positioning of individuals near transformers are random and irregular. Therefore, this study simulates the effects of electromagnetic radiation from three-phase transformers on the heart and magnesium alloy stent in patients at various locations under and around the transformer, based on realistic daily-life conditions for stent implant recipients. Numerical results indicate that under the specific simulated conditions, the magnetic flux density, induced electric field in the cardiac tissues, and the Ampere force acting on the magnesium alloy stent all remain below the established safety limits. However, given potential individual variations and extreme scenarios, further validation is recommended before generalizing these findings to all real-world situations.
